# Phytochemical variation in treetops: causes and consequences for tree-insect herbivore interactions

**DOI:** 10.1007/s00442-018-4087-5

**Published:** 2018-02-23

**Authors:** Jörn. S. Lämke, Sybille B. Unsicker

**Affiliations:** 1 Institute for Biochemistry and Biology, University of Potsdam, Karl-Liebknecht-Str. 24-25, 14476 Potsdam, Germany; 2 Department of Biochemistry, Max Planck Institute for Chemical Ecology, Hans-Knöll Str. 8, 07745 Jena, Germany

**Keywords:** Chromatin-based mechanisms, Genotypic variation, Insect herbivore, Phenotypic plasticity, Tree defence

## Abstract

The interaction of plants and their herbivorous opponents has shaped the evolution of an intricate network of defences and counter-defences for millions of years. The result is an astounding diversity of phytochemicals and plant strategies to fight and survive. Trees are specifically challenged to resist the plethora of abiotic and biotic stresses due to their dimension and longevity. Here, we review the recent literature on the consequences of phytochemical variation in trees on insect–tree–herbivore interactions. We discuss the importance of genotypic and phenotypic variation in tree defence against insects and suggest some molecular mechanisms that might bring about phytochemical diversity in crowns of individual trees.

## Introduction

Plants and insects coevolved since more than 350 million years (Whitney and Glover 2013) and during this time plants have developed an enormous diversity of chemical defence compounds. An arms race between insects and plants is thought to be the main driver of diversification in plant defence chemistry (Ehrlich and Raven 1964). Trees, as long-lived woody perennials, are dominant components of terrestrial ecosystems and they host an enormous diversity of insects (Basset et al. 2012). Their longevity, their size, their architecture and the formation of wood make the appearance of trees very different from herbaceous plant species. A survey of leaf herbivory across all major plant lineages revealed that compared to herbaceous plants, woody species experience 60% more herbivory (Turcotte et al. 2014). How can individual trees withstand these loads of herbivores and the amount of concomitant damage throughout their lifetime of sometimes hundreds of years? They have evolved physical barriers such as spines and thorns as well as tough, lignified leaves and on top of this they produce a large diversity of carbon-based phytochemicals such as phenolics and terpenoids as defences against their attackers. Additionally, there are vertical and horizontal gradients in abiotic conditions in treetops that can promote variation in tree defence chemistry which in turn can affect insect herbivore performance. In the eye of a tiny herbivorous insect, the treetop of a single tree is not just a homogenous predictable habitat but rather a heterogeneous and often inhospitable environment. Phytochemical diversity in treetops has the potential to shape insect community diversity and population structure as recent studies in woody species within the tropical genus *Piper* convincingly showed (Glassmire et al. 2016; Richards et al. 2015). In the light of this, it seems surprising that both the differences in the phytochemical composition within a tree crown, as well as the elucidation of potential mechanism maintaining phytochemical variation, has received little attention (Table 1).

**Table 1:** Studies on phytochemical variation in different tree species

Tree species	Source of variation	Tree age	Number of genotypes	Location	Approach	Canopy layer	Chemical trait	Herbivore species	Measures of higher trophic level consequences	References
*Alnus glutinosa*	Drought, herbivory	Immature	Multiple	G	E		VOC	*Monsoma pulveratum*	Experimental herbivory	Copolovici et al. (2014)
*Betula pendula*	Herbivory	9–11 years	Multiple	F	E	Different	Phenolics	*Lymantria dispar*	Larval fitness and resistance against pathogen	Martemyanov et al. (2012)
*Betula pubescens*	Genotype, environment, fertilization	Mature	Multiple	C	E	Different	Protein-bound amino acids, phenolics, phenoloxidase activity	*Epirrita autumnata*	Larval development	Haviola et al. (2012)
*Betula pubescens*	Herbivory	Mature	Multiple	F	E	Different ≤ 3 m	N, phenolics	*Epirrita autumnata*	Larval performance	Kaitaniemi et al. (1998)
*Eucalyptus globulus*	Genotype, geographic range	15 years	Multiple	C	D	Upper (felled trees)	Phenolics, CT and oils (1,8-cineole); N, C, H; formulate phloroglucinol compound	Selected arthropods	Arthropod abundance assessed via leaf symptoms	Barbour et al. (2009)
*Eucalyptus globulus*	Genotype	Immature	Multiple	C	D		Terpenes, foliar wax	None	Arthropod abundance	Glassmire et al. (2016)
*Eucalyptus melliodora E. sideroxylon*	Phenotypic mosaicism	Mature	One	F	D	Different	Terpenes, formylated phloroglucinol compounds	None	None	Padovan et al. (2012)
*Eucalyptus melliodora, E. sideroxylon*	Phenotypic mosaicism	Mature	One	F	D	Upper	?	None	None	Padovan et al. (2015)
*Fagus crenata*	Space, season	?	?	F	D	Different	Phenolics, C, N	None	Leaf area damaged	Yamasaki and Kikuzawa (2003)
*Fagus sylvatica*	Herbivory	Immature	Multiple	OP	E		VOC	*Lymantria dispar*	None	Gossner et al. (2014)
*Malus × domestica*	Rainfall, temperature, relative humidity	?	?	P	D	Mid	VOC	None	None	Vallat et al. (2005)
*Malus × domestica*	Diurnal rhythm	Immature	One	C	D		VOC	None	None	Giacomuzzi et al. (2017)
*Malus × domestica*	Herbivory	Immature	Multiple	C	E		VOC	*Epiphyas postvittana*	Parasitoid attraction	Suckling et al. (2012)
*Malus × domestica*	Drought	Immature	Multiple	G	E		Phloridzin, phloretin, sugars	*Spodoptera littoralis*	Caterpillar feeding preference	Gutbrodt et al. (2012)
*Picea abies*	Genotype, environment, herbivory	Mature	Multiple	C	D	Lower	Phenolics	*Adelges spp., Sacchiphantes spp.*	Gall abundance	Axelsson et al. (2015)
*Pinus edulis*	Herbivory, season	Mature	Multiple	F	D	Lower	VOC	*Lophocampa ingens*	Leaf area damaged	Trowbridge et al. (2014)
*Pinus pinaster, P. radiata*	Herbivory, nutrients	?	Multiple	P	E	?	N, terpenes, tannins, phenolics, activity of peroxidase, polyphenoloxidase, chitinase and trypsin inhibitor	*Thaumetopoea pityocampa*	None	Lombardero et al. (2016)
*Pinus sylvestris*	Herbivory	8–25 years (2 m high)	Multiple	F	E	?	Phenolics, CT and oils; N, carbon, hydrogen; formylated phloroglucinol compounds	*Diprion pini*	Sawfly fitness	Roitto et al. (2009)
*Pinus sylvestris*	Herbivory	Immature	Multiple	OP	E		VOC	*Hylobius abietis*	None	Heijari et al. (2011)
*Populus alba, P. tremula, hybrid*	Hybridization, genetic architecture	Mature, immature	Multiple	C, F	D	?	phenylpropanoids	None	None	Caseys et al. (2015)
*Populus deltoides* × *P. nigra, P. laurifolia* × *P. nigra, P. laurifolia* × *P. nigra*	Drought	Immature	One	G	E		?	None	None	Raj et al. (2011)
*Populus fremontii, P. angustifolia, hybrid*	Ontogeny	Mature and immature	Multiple	C, F	D	Different	N, CT, salicinoids	None	None	Rehill et al. (2006)
*Populus fremontii, P. angustifolia, hybrids*	Herbivory, ontogeny, genotype	18-year old	Multiple	C	E	Lower, mid	N, CT, salicinoids	*Chrysomela confluens*	None	Holeski et al. (2012)
*Populus fremontii, P. angustifolia, hybrids and backcrossings with P. angustifolia*	Genotype	11 years	Multiple	C, F	D		Phenolics, N	None	Arthropod composition	Bangert et al. (2006)
*Populus nigra*	Drought, fertilization	Immature	One	C, G	E		Salicinoids	*Lymantria dispar, Orgyia leucostigma*	Larval growth	Hale et al. (2005)
*Populus nigra*	Herbivory, diurnal rhythm	Immature	One	C	E		VOC	*Lymantria dispar, Laothoe populi*	Experimental herbivory	Clavijo Mc Cormick et al. (2014a)
*Populus nigra*	Herbivory	Immature	Multiple	C	E		VOC, defence hormones	*Lymantria dispar*	Experimental herbivory	Clavijo Mc Cormick et al. (2014a)
*Populus nigra*	Herbivory, ontogeny	Mature	Multiple	F	E	Lower	Phenolics, defence hormones	*Lymantria dispar*	Experimental herbivory	Boeckler et al. (2013)
*Populus tremula*	Geographic structure	Immature	Multiple	C	D		Non-targeted metabolome	None	Herbivore community structure	Bernhardsson et al. (2013)
*Populus tremula*	Genotype, environment	Immature	Multiple	C	D		Phenolics	None	Arthropod community structure	Robinson et al. (2015)
*Populus tremula × tremuloides*	Ozone, herbivory	Immature	Two	C	E		VOC	*Phyllobius piri, Epirrita autumnata*	None	Blande et al. (2007)
*Populus tremuloides*	Frost	?	Multiple	F	D	Lower, mid, upper	Sugars, N, P, S, K, Ca, Mg, Fe, salicinoids, CT	None	None	St Clair et al. (2009)
*Populus tremuloides*	Genotype, herbivory	?	Multiple	F	D	?	N, CT, salicinoids	*Malacosoma disstria*	Defoliation estimates	Donaldson and Lindroth (2008)
*Populus tremuloides*	Genotype, tree age	0 to > 20 years	Multiple	F	D	?	Salicinoids, CT, starch, sugars, N	None	None	Donaldson et al. (2006)
*Populus tremuloides*	Frost, genotype	Immature	Multiple	C, G	E		N, CT, salicinoids	*Chaitophorus stevensis*	Aphid population growth	Rubert-Nason et al. (2017)
*Populus tremuloides*	Genotype, nutrients, herbivory	Immature	Five	OP	E		N, CT, salicinoids	*Lymantria dispar*	Larval survival and performance	Rubert-Nason et al. (2015)
*Populus tremuloides*	Ontogeny	Mature	Multiple	F	D	Mid	N, P, sucrose, starch, CT and salicinoids	None	None	Smith et al. (2011)
*Populus tremuloides*	Herbivory	Immature	Multiple	S	E		N, CT, salicinoids	*Lymantria dispar*	Percent defoliation	Donaldson and Lindroth (2007)
*Populus tremuloides, Betula papyrifera*	CO_2_, ozone	10–12 years	Multiple	C	E	Lower, upper	N, sugars, starch, lignin, CT, salicinoids	None	None	Couture et al. (2017)
*Populus tremuloides, Betula papyrifera*	Climate warming	Immature	Multiple	F	E		N, soluable sugars, starch, CT, salicinoids	*Malacosoma disstria*	Feeding behavior, larval performance	Jamieson et al. (2015)
*Quercus castanea*	Genotype, herbivory	Mature	Multiple	F	D	Lower, mid, upper	Tannins, suluable phenolics, proanthocyanidins	None	Leaf area damaged	Maldonado-Lopez et al. (2015)
*Quercus ilex*	Herbivory	Immature	Multiple	C	E		VOC	*Lymantria dispar*	None	Staudt and Lhoutellier (2007)
*Quercus robur*	Herbivory	10–12 years	Multiple	F	E	?	VOC, leaf pigments	*Lymantria dispar*	Leaf area damaged	Copolovici et al. (2017)
*Quercus robur*	Altitude, temperature, herbivory	Mature	Multiple	F	D	Lower	Phenolics, nutrients	None	Leaf damage by chewers, miners, gallers	Abdala-Roberts et al. (2016)
*Tilia cordata*	Herbivory, height within tree	22 years	Multiple	P	D,E	Lower, upper, inner	N, sugars, tannins	*Popillia japonica*	Beetle feeding preference and performance	Rowe and Potter (1996)

The “experimental approach” is classified in D (descriptive) and E (experimental); “location” is categorized in C (climate chamber), G (greenhouse), F (field), OP (outdoor pots), P (plantation) amd S (screenhouse). Canopy layers in immature trees are not specified. Whenever the specific information in the reference is missing, this is denoted with a questionmark

Abbreviations for chemical traits: *VOC* volatile organic compound, *CT* condensed tannins, *N* nitrogen, *C* carbon, *P* phosphorous, *H* hydrogen, *S* sulfur, *K* potassium, *Ca* calcium, *Mg* magnesium, *Fe* iron

**Fig. 1:**
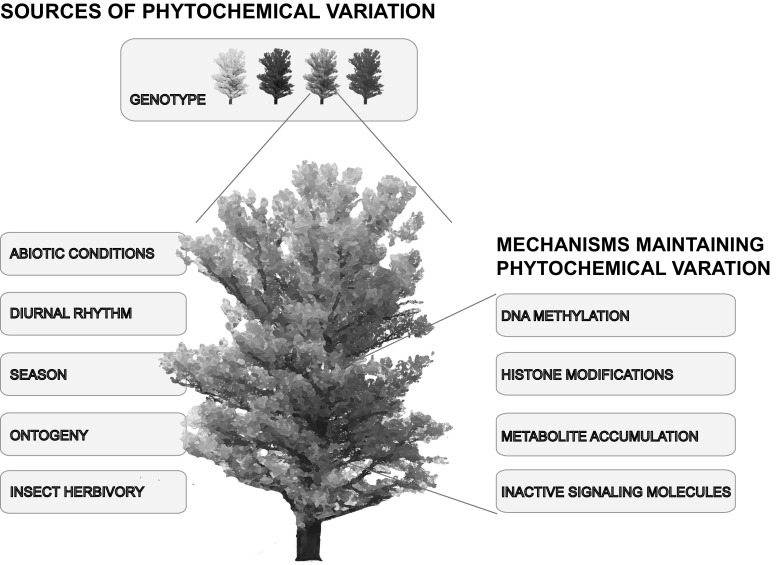
Topics covered in this review article. The diagram depicts sources of phytochemical variation in trees and possible molecular mechanisms maintaining this variation in treetops

Here, we review the recent literature of the last 15 years on causes and consequences of intra-specific variation in tree defence chemistry against insect herbivores aboveground. Recent findings on the role of abiotic conditions, tree genotype, spatial and temporal patterns, ontogeny and herbivore feeding for tree phytochemical variation are summarized (Fig. 1). In this manuscript, we want to specifically emphasize the phytochemical variation within treetops of old-growth trees, and the consequences for insect herbivores, as the vertical dimension of trees has so far almost been neglected in studies on tree defence chemistry (Table 1). In the second part of this review, we outline different molecular mechanisms that contribute to the maintenance of phytochemical variation in plants (Fig. 1). The recent literature from mostly herbaceous species is used to suggest molecular mechanisms responsible for the variation in tree defence chemistry against insect herbivores. In a final chapter, we point out the lack of knowledge in the mechanistic understanding of tree defence against insect herbivores under natural conditions and suggest an interdisciplinary research approach to study the ecology of tree-insect interactions in the future.

## The tree genotype determines intra-specific variation in phytochemistry

Intra-specific genotypic variation in trees is known to be a major driver of phenotypic plasticity that can also shape arthropod community structures via genotypic effects on variation in tree defence chemistry (Donaldson and Lindroth 2007; Whitham et al. 2006, Bernhardsson et al. 2013). Studies in poplar trees have shown that intra-specific genotypic variation has strong effects on the concentration of compounds in the two major groups of phenolic defences, condensed tannins and salicinoids (e.g., Donaldson and Lindroth 2007). Genotypic effects on tree phytochemistry were, e.g., also shown in studies on willow (Barbour et al. 2015), *Eucalyptus* (Barbour et al. 2009; Gosney et al. 2017) and birch (Haviola et al. 2012). The phytochemistry of trees has been suggested to be the intermediate link between tree genes and the arthropods associated with trees by the genetic similarity rule (Bangert et al. 2006). However, empirical studies have shown that the tree genotype is not always the best predictor for arthropod community composition and insect herbivore feeding patterns. In a study by Maldonado-Lopez et al. (2015) on the relationship between red oak genetics, phytochemistry and damage patterns by two herbivorous feeding guilds, leaf chewers and leaf miners, only damage by the latter was explained by genetics and tree chemistry. In Norway spruce galling aphid communities were not related to tree phytochemical profiles and tree genetics only affected the abundance of galls within one taxonomic group but not the other (Axelsson et al. 2015). A recent study comparing 100 naturally growing adult oak trees (*Quercus robur*, *Q. petraea*) found no evidence for genotype effects on arthropod communities but chemical traits as potential links between tree genetics and arthropod community structure were not explicitly investigates in this study (Gossner et al. 2015).

## Abiotic conditions affect the tree defence chemistry

Certainly a main driver of phytochemical variation in trees is the abiotic environment which in itself can vary dramatically throughout the lifetime of a tree and even through the course of 1 day. A number of recent common garden and laboratory studies investigated the impact of abiotic conditions such as rainfall, humidity, nutrient availability and temperature on tree defence chemistry (e.g., Jamieson et al. 2015; Vallat et al. 2005). Together with studies looking at phytochemical variation in trees in response to climate change scenarios with, e.g., increases in temperature, O^3^, CO_2_, as well as more frequent drought and frost periods [recently reviewed by Lindroth (2010) and Jamieson et al. (2012)], a picture emerges where the phytochemical composition is heavily influenced not only by the genetic make-up of a given species, but also by these non-intrinsic abiotic factors (Blande et al. 2007; Copolovici et al. 2014; Couture et al. 2017; Gutbrodt et al. 2012; Hale et al. 2005). The phytochemistry of young *Populus tremuloides* trees substantially changed in response to experimental vernal freezing (Rubert-Nason et al. 2017) with decreased concentrations of condensed tannins and slightly increased levels of phenolic glycosides in the foliage of frost-stressed trees as compared to control trees. In naturally growing mature *P. tremuloides* trees, however, vernal freezing induced only changes in phenolic glycoside levels (St Clair et al. 2009). A recent study by Abdala-Roberts et al. (2016) suggests that temperature is the most important factor explaining variation in the defence chemistry of mature pedunculated oak (*Q. robur*) trees occurring at different altitudes in Northern Spain. In this study, the foliar concentrations of phenolic compounds (rutin, gallic acid and catechin) significantly increased with decreasing mean annual temperatures of 4 °C across an elevation gradient of around 800 m.

## There are strong temporal and ontogenetic patterns in tree defence chemistry

The phytochemical composition in trees can also strongly vary over time (Yamasaki and Kikuzawa 2003) and diurnal rhythms of, e.g., tree volatile emission (Clavijo McCormick et al. 2014a; Giacomuzzi et al. 2017; Trowbridge et al. 2014) as well as seasonal changes in carbon-based defence compounds were documented (Gripenberg et al. 2007; Holeski et al. 2012). The chronologically oldest branches in a tree, i.e., closest to the root crown will exhibit the youngest phenotype whereas the most distant shoots at the outer rim of the tree crown display the more mature phenotype. Kearsley and Whitham (1998) termed this counterintuitive phenomenon of within-tree phenotypic plasticity the “developmental stream”. Ramets within the crown of one tree genotype can, thus, vary significantly in their phytochemical profiles (Rehill et al. 2006; Smith et al. 2011) and even within these ramets an ontogenetic gradient in phytochemistry can occur (Boeckler et al. 2013).

## Insect herbivory is a major source of phytochemical variation in trees

One of the main reasons for the observed phytochemical variation within tree species might very well be explained by differences in individual biotic interactions with pathogens and herbivores (vertebrates and invertebrates). Unlike simplified single species interactions studied in the greenhouse and in the lab, naturally growing trees of all age classes are simultaneously attacked by numerous insects and pathogens. This induces variable levels of damage, ranging from losses of a few leaves to complete defoliation. Attack by an insect herbivore induces rapid local and systemic responses by de novo synthesis and relocation of defence compounds such as phenolics or terpenoids. Phenolics in tree leaves can make up to a quarter of the leaf dry weight as in the case of condensed tannins and salicinoids in aspen (Donaldson and Lindroth 2008; Donaldson et al. 2006). These compounds are constantly present in tree tissues and thus termed constitutive defences just like terpenoids in coniferous resins are. Insect feeding, however, can induce an increase in the concentration of these phytochemicals. The induction of phenolics in trees is dependent on the tree species, the genotype and the attacking insect herbivore species. In poplar for instance, only a few studies have shown the induction of salicinoids (phenolic glycosides), a major group of phenolic defences (Rubert-Nason et al. 2015), whereas other studies did not see induction after herbivore attack at all (Boeckler et al. 2013). Insect herbivory also induces a change in the composition of volatile organic compounds (VOCs) released from trees. Upon gypsy moth (*Lymantria dispar*) caterpillar feeding young black poplar (*Populus nigra*) trees increase their emission of VOCs by more than 20-fold and the herbivore-induced blend qualitatively differs from the volatiles released from non-damaged control trees. Minor nitrogenous compounds (aldoximes and nitriles) are only emitted by the trees when they are attacked by herbivores (Clavijo McCormick et al. 2014b) and the composition of herbivore-induced black poplar VOCs also varies in response to different herbivore species (Unsicker et al. 2015). Variation in tree VOC emission due to insect herbivore feeding has been reported in a number of tree species such as pine (Heijari et al. 2011; Trowbridge et al. 2014), oak (Copolovici et al. 2017; Staudt and Lhoutellier 2007), alder (Copolovici et al. 2014), beech (Gossner et al. 2014), apple (Suckling et al. 2012) and willow (Yoneya et al. 2010). Changes in VOC emission upon insect herbivore damage are not restricted to the locally damaged sites but also occur in non-damaged adjacent foliage in apical direction (Clavijo McCormick et al. 2014b). Under field conditions in old-growth black poplar trees, however, this systemic induction of herbivore-induced VOCs was not significant (Unsicker unpublished data). Besides producing defence chemicals immediately upon insect herbivore damage, trees are also able to respond to severe defoliation by increasing their defence in the next growing season. This phenomenon termed “delayed-inducible resistance” has been shown for a number of mainly deciduous tree species (e.g., Haukioja 1991; Kaitaniemi et al. 1998; Martemyanov et al. 2012) but also conifers with inconsistent results (Lombardero et al. 2016; Roitto et al. 2009).

## Phytochemical variation in treetops: the overlooked vertical dimension

It may seem trivial to specifically point out here that all abiotic and biotic variables influencing intra-specific variation in tree phytochemistry can also cause phytochemical variation within the treetop of a single tree. Under natural conditions, the abiotic conditions in treetops can vary drastically along the vertical and horizontal axis. The outer part of the tree crowns experiences very different levels or irradiation, wind speed, temperature and humidity than the innermost crown areas. As a consequence, microclimatic conditions within trees can be highly variable. Additionally, spatial variation in arthropod abundance and insect herbivore feeding in tree crowns have been observed (Robinson et al. 2012, Basset et al. 2003; Rowe and Potter 1996; Unsicker and Mody 2005; Yamasaki and Kikuzawa 2003) and thus it seems intuitively logical, that there must also be a large spatial component in the variation of tree defence chemistry within the treetop of a single tree. Unfortunately, most studies on tree defence chemistry, specifically the ones with experimental approaches, have been performed in small, immature trees likely due to the difficulties in accessing large old-growth trees (Barker and Pinard 2001). To our knowledge, there is hardly any study that focused specifically on phytochemical variation in different layers of large, mature trees (Table 1).

## Molecular mechanisms of phytochemical variation in tree species and individual treetops

The diversification of defence compounds and defence strategies within tree species is largely based on genetic variation. The mechanisms creating the substrate for this evolutionary change are diverse and a detailed review of these is beyond the scope of this article (for a review, see, e.g., Chen et al. 2013). One prominent mechanism for creating genetic diversity is the duplication of genes or, more prominent in plants, whole genome duplications (Panchy et al. 2016). Most duplicated genes are lost in the course of evolution (Lynch and Conery 2003) but when they are retained, they can acquire new functions. One illustrative example for this is the massive diversification of compounds within the group of terpenoids. Currently, more than 30.000 different terpenes are known (Keeling and Bohlmann 2006). Here, different terpene synthases (mono-, sesqui-, and diterpene synthases), which apparently evolved through repeated duplication followed by functional diversification, produce an amazingly diverse array of terpene backbones (Zapata and Fine 2013). Interestingly, the diversification in the group of terpenoids might be due to different mechanisms in mono- and dicot species (Boutanaev et al. 2015). Species hybridization **c**an furthermore increase the phytochemical diversity in trees (Caseys et al. 2015) in a local context as could be envisioned for the local accumulation of advantageous single nucleotide polymorphisms (SNPs) (Bernhardsson and Ingvarsson, 2012). In trees, the above-mentioned mechanisms do not only lead to a diversification of compounds, but also ultimately, and possibly more importantly from an ecological perspective, shape community compositions of a given habitat (Whitham et al. 2006) and additionally provide the basis for new species interactions at the ecosystem level (Benfey and Mitchell-Olds 2008).

The importance of the above-described mechanisms in creating species diversity on an evolutionary time scale cannot be overestimated. For a single tree, faced with the challenge of responding to myriads of attackers throughout its lifetime, however, the phytochemical diversity created in the past is a mere platform to act and survive in the present, using the arsenal provided by its (lifetime-wise) largely invariant genome (Sarkar et al. 2017). However, somatic mutations (alterations in the genetic information that is not transmitted to the next generation) might, in specific cases, play a role for phenotypic diversity within an individual tree as in the case of mosaic trees within the genus *Eucalyptus* (Padovan et al. 2012, 2015).

Given the vertical and horizontal dimension of mature tree crowns, the challenges one crown area faces might be very different to what another crown area tackles at the same time. Consequently, the heterogeneity of influential variables may lead to local adaptations in different parts of the treetop.

In the following, we will review potential mechanisms leading to intra-crown (treetop) diversity in phytochemistry. The sensing of a local challenge (e.g., insect herbivory) provides informational value for the tree that might be relevant for other parts of the tree as well. Transmission of this information requires efficient and fast means of communication between both the affected, as well as the (yet) unaffected tissues, which can be realized, e.g., by VOC emission (Heil and Karban 2010). As mentioned earlier, trees emit specific blends of VOCs upon herbivore attack but interestingly, this signal is only emitted as long as there is actual feeding (Clavijo Mc Cormick et al. 2014a). Herbivore-induced VOCs, thus, signal a potential threat in the future and prime non-damaged tissues for a faster and stronger response, e.g., upon a second herbivory event (Frost et al. 2008). This raises the question how this perceived information is stored and then only transferred into a chemical defence response when, e.g., insect herbivore attack happens. Mechanistically, this requires several steps: the information needs to be spread to receivers and be decoded (e.g., VOCs emitted upon herbivory need to be sensed and linked to a response). After decoding the information, some kind of memory of this information needs to be established and this memory then alters the response when a specific stress (e.g., herbivory) recurs. Here, different (and certainly nonexclusive) mechanisms to store information locally have been proposed. These range from an increase in inactive signalling compounds like signalling kinases (Beckers et al. 2009), which, once activated by a specific stimulus, lead to a massive amplification of signalling and hence a potentially quicker and stronger response. Another possibility is the accumulation of specific metabolites (Navarova et al. 2012), which are either directly involved in defence or which serve as signalling molecules that can be released once stress recurs. A widely observed pattern in primed plant responses are alterations in transcriptional activity, where a primed transcriptional response is different from the transcriptional response when stress is encountered for the first time (Hilker et al. 2016). When altered transcriptional responses are observed, chromatin modifications offer a mechanistically intuitive way of modulation. In the nucleus, DNA is organized in a structure called chromatin (all nuclear DNA and associated proteins like histones); modifications to histones or DNA either directly or indirectly regulate the accessibility of genomic loci and either facilitate or restrict transcriptional activity. Indeed, chromatin was long viewed as an interface between the environment and the genome. In genetically identical ramets of poplar, for example, globally altered DNA-methylation patterns depending on growth history were described (Raj et al. 2011). In herbaceous plants, recurring stress lead to altered levels of histone modifications at stress relevant loci, which correlated with altered transcriptional responses when stress recurred (Ding et al. 2012; Jaskiewicz et al. 2011; Lämke et al. 2016). These works established histone 3, lysine 4 hypermethylation as a potential memory mark that might be instructive for altered transcriptional activity when loci are re-activated upon a second stress. Of note, this chromatin modification persisted long after the initial transcriptional activity ceased and hence might store the perceived information (Conrath et al. 2015; Lämke and Baurle 2017). In case of priming within the tree crown, this scenario suggests that priming might lead to different chromatin states within the crown, which then allow for the modulation of (transcriptional) responses when a stress either spreads or recurs, leading to locally different phytochemical responses to the same challenge. Indeed, alterations in transcriptional responses are observed upon priming by volatiles and subsequent challenge (Frost et al. 2008). It seems reasonable to assume that trees use chromatin-based mechanisms extensively to store perceived information within the tree crown to allow for an adapted response. We are currently lacking a clear picture of both the extent as well as the duration of chromatin based memory in trees. Given the very long life span and sheer size of a tree, resulting both in the constant need to adapt to the changing local environment and the highly informative value of previous stress exposure, it seems very plausible that trees use chromatin-based means extensively to constantly adapt and be prepared for future challenges (Bräutigam et al. 2013).

## Critical remarks and future directions

In this article, we outlined different sources of phytochemical variation within tree species and individual treetops and suggested mechanisms at the molecular level to maintain this variation.

An obvious drawback in the studies on tree defence chemistry and the consequences for insect herbivores is that they are limited to a narrow range of tree species or genera (i.e., oak, poplar, willow, pine, eucalyptus, birch) and within those only a few or single tree genotypes. Furthermore, most experimental studies investigating tree defence mechanisms are performed under controlled greenhouse or laboratory conditions with immature trees, raising the question whether the results from these studies allow us to deduce generalities and make predictions also for mature trees under natural conditions. Experimentally applied abiotic and biotic stresses are mostly inflicted singly and only rarely are trees under laboratory conditions exposed to real-world scenarios with, e.g., simultaneously occurring biotic and abiotic stresses. Even under field conditions, the majority of studies on tree defences investigate younger trees of reasonable height, as it is certainly challenging, if not impossible, to obtain samples for phytochemical analysis representing the entire treetop of a large old-growth trees. Furthermore, field studies are mostly descriptive and rarely imply experimental approaches with modern molecular methods. Well-replicated experimental approaches within treetops of old-growth trees are very demanding and likely restricted to sites with canopy cranes, canopy walkways or trees accessible with, e.g., the single rope climbing technique. Despite these difficulties, we urgently need the synthesis of field based experiments in old-growth trees with experimental approaches using modern molecular techniques to reveal the causes and consequences of phytochemical variation in trees for tree-insect-herbivore interactions. Here, “genome-enabled field biologists” (Baldwin 2012) with a fascination for climbing trees should step up to the plate.
